# Herbicide applications increase greenhouse gas emissions of alfalfa pasture in the inland arid region of northwest China

**DOI:** 10.7717/peerj.9231

**Published:** 2020-05-25

**Authors:** Lina Shi, Yarong Guo, Jiao Ning, Shanning Lou, Fujiang Hou

**Affiliations:** State Key Laboratory of Grassland Agro-ecosystems, Key Laboratory of Grassland Livestock Industry Innovation, Ministry of Agriculture and Rural Affairs, Engineering Research Center of Grassland Industry, Ministry of Education, College of Pastoral Agriculture Science and Technology, Lanzhou University, Lanzhou, China

**Keywords:** Herbicides, Weeds, Control, Greenhouse gases, Alfalfa

## Abstract

Herbicides are used to control weeds in agricultural crops such as alfalfa (*Medicago sativa* L.), which is a forage crop. It is unclear what, if any, effect herbicides have on greenhouse gas (GHG) emissions when used on alfalfa. Our study was conducted in 2017 and 2018 to investigate the effects of two herbicides (Quizalofop-p-ethyl, QE and Bentazone, BT) on methane (CH_4_), carbon dioxide (CO_2_) and nitrous oxide (N_2_O) emissions from soil planted with alfalfa. QE is used to control grasses and BT is used for broadleaf weed control. Soil CO_2_ emissions and soil uptake of CH_4_ increased significantly in both years following the QE and BT treatments, although CO_2_ emissions differed significantly between the trial years. N_2_O emissions decreased relative to the control and showed no significant differences between the trial years. The application of QE and BT on alfalfa resulted in a significant increase in CO_2_ emissions which contributed to a significant increase in GHG emissions. The application of QE influenced GHG emissions more than BT. We demonstrated the potential effect that herbicide applications have on GHG fluxes, which are important when considering the effect of agricultural practices on GHG emissions and the potential for global warming over the next 100 years.

## Introduction

Large amounts of agrochemicals, including herbicides, insecticides and fungicides, are used to control agricultural threats like weeds, pests and pathogens (Dennis et al., 2018). The average worldwide use of agricultural pesticides amounted to 2.63 kg·ha^−1^ in 2017. However, the average use in China peaked at 13.07 kg·ha^−1^ in the same year (The Food and Agriculture Organization, 2017). We predict that the use of herbicide in crop production will continue to increase globally, particularly in developing countries, due to a shortage of manual labor for hand weeding that affects their crop yields (Gianessi, 2013). Herbicides can quickly and effectively inhibit the growth of weeds and increase crop yield, but also indirectly affect the flux of soil GHG by changing vegetation-soil interactions (Ahmed, Chauhan & Humphreys, 2013). GHG, particularly CH_4_, CO_2_ and N_2_O, have aroused considerable concern in recent decades because of the acknowledged contribution of these gases to global warming. Agricultural soils are major anthropogenic sources of GHG and account for approximately 60% of CH_4_, 15% of CO_2_ and 61% of N_2_O emissions (IPCC, 2013). Herbicides influence the interspecific competition between crops and weeds and affects the composition of the vegetation (Kökten & Tükel, 2009), which affects soil moisture and temperature (Leuschner & Lendzion, 2010). Herbicides may change the structure and function of the soil’s microbial communities by affecting their levels of enzymatic activity (Crouzet et al., 2010). This, in turn, impacts the activity of fungi and bacteria associated with the decomposition of plant matter and ultimately all soil GHG emissions (Wang et al., 2017).

Herbicidal research has focused on the mode of action (Mithila et al., 2011), efficacy (James et al., 2018; Karimmojeni et al., 2013), and resistance of plants to herbicides (Owen, Martinez & Powles, 2014*; Yu & Powles, 2014*). Relatively little attention has been paid to the impact of herbicide use on GHG emissions to date. Gianessi (2013) demonstrated that the use of herbicides reduced GHG emissions by reducing the fuel consumption of traditional farming machinery that would otherwise be used to manage weeds. Jiang et al. (2015) demonstrated that the application of Butachlor and Bensulfuron-methyl in winter wheat fields reduced N_2_O emissions. The application of these herbicides to irrigated rice fields significantly reduced CH_4_ and N_2_O fluxes. Although single applications of Bensulfuron-methyl and Pretilachlor reduced GHG emissions, the combination of these two herbicides was shown to increase the emissions of N_2_O and CH_4_ (Das, Ghosh & Adhya, 2011). Oyeogbe et al. (2017) demonstrated that strategic nitrogen fertilizer use and integrated weed management reduced GHG emissions. Chemicals play an important role in GHG emissions and mitigation, but these effects are still not well known in grassland ecosystems as the majority of research has focused on cropland ecosystems. Most grasslands studies have concentrated on annual grass crops rather than perennial and legume crops. Legumes (e.g., alfalfa) are important crops owing to their yield-forming potential and high protein value (Staniak & Harasim, 2018). Alfalfa is widely grown all over the world and is the largest forage crop sown in China. The majority of these crops are grown in the northwest arid region of China, including Gansu, Xinjiang, Ningxia, and Inner Mongolia. Alfalfa protects the ecology of the northwest arid region of China and contributes to the development of animal husbandry (Feng et al., 2016). It covers a total area of 4,745 × 10^3^ ha in China (Wei et al., 2018) and herbicide application is necessary to produce a maximum yield.

Quizalofop-p-ethyl (QE) is a commonly used selective herbicide that systemically protects leguminous forage from grass weeds (Fan, Sun & Fan, 2001). Bentazone (BT) is a post-emergence herbicide used for the selective control of broadleaf weeds and sedges in beans, rice, corn, peanuts and other crops (Ribeiro et al., 2011). Although these two herbicides are widely used in alfalfa crops throughout the world, their effects on GHG emissions in soil planted with alfalfa remains unknown. The aims of this study were: (1) to investigate the effect of the application of QE and BT on GHG emissions of alfalfa pasture and (2) to analyze the mechanisms of effect these chemicals had on GHG emissions and to provide a best-practice reference for the management of alfalfa for forage production.

## Materials and Methods

### Site description

Field trials were conducted from 2017 to 2018 at the Linze Grassland Agriculture Station of Lanzhou University, located in the core area of the Heihe Oasis in Hexi Corridor, Northwest China (100°02′E, 39°15′N; 1,390 m above sea level). The area has a temperate continental climate with distinct seasons that include long, cold winters, short, hot summers, rapid warming in the spring and slow cooling in autumn. The annual average temperature is 7.7 °C, with a ≥0 °C accumulated temperature of 3,026 °C. The annual average precipitation is 118.4 mm and the evaporation rate is 1,830.4 mm. More than 70% of the precipitation occurs from May to September. Specialized intensive cropping production systems and extensively integrated crop-livestock production systems dominate the agricultural practices of this region (Hou et al., 2008).

*Plantago asiatica* L., *Agropyron cristatum* Gaertn., *Digitaria sanguinalis* Scop., *Chenopodium glaucum* L., *Portulaca oleracea* L., *Setaria viridis* Beauv., *Echinochloa crusgalli* Beauv., *Suaeda glauca* Bunge and *Ixeris denticulata* Stebbins were the dominant weeds found in the study area. Many of these weeds are annual and seeds begin heading in the latter half of August and mature after August and September.

### Experimental design

Our experiment was randomized with three replicates per treatment. Each test plot (8 m × 10 m) was separated by a guarding row. Alfalfa (var. Algonquin) was sown randomly in three 50 m × 60 m plots in August of 2012 and 2013, respectively. On September 5, 2017 and August 12, 2018, two herbicides (QE and BT) were applied using a knapsack sprayer to the alfalfa trial plots using their recommended application rates (QE, 0.9 L·ha^−1^ in 4.5 L water·ha^−1^; BT, 30 L·ha^−1^ in 150 L water·ha^−1^). Water with no herbicide was applied to the control plots (CK).

### GHG, soil, and aboveground biomass sampling and analysis

Measurements of CH_4_, CO_2_ and N_2_O, the three principal GHGs, were collected using three static opaque chambers on each plot. The aboveground biomass was first cut to eliminate the inference of alfalfa photosynthesis and respiration on the gas samples. Samples were taken between 09:00–11:00 AM (Liu et al., 2017) for 3 weeks after the herbicides were applied. Samples were taken on day 2, day 4, day 6, day 10 and day 15 in 2017 and on day 2, day 4, day 6, day 8, day 10, day 15 and day 20 in 2018. Each chamber measured 30 cm × 30 cm × 30 cm and was constructed to be gas-proof by fitting the base of its walls into a water-filled collar inserted 30 mm into the soil. Each chamber was equipped with a battery-operated fan to mix the gases. Gas samples were collected through a three-way tap using a 50 ml syringe and were then transferred to a 500 ml aluminum foil gas collection bag. Four gas sample bags were collected per each sampling process and samples were taken 0, 10, 20 and 30 min after placing the chamber to represent the daily average GHG flow. Approximately 300 ml of the gas from each gas sample was brought back to the laboratory for analysis. The temperature inside the chamber and the soil temperature (ST, °C at a depth of 50 mm) was measured simultaneously during gas sampling.

A CH_4_/CO_2_ analyzer (DLT-100, Model No. 908-0011-0001) was used to determine the concentration of CH_4_ and CO_2_ and a CO/N_2_O analyzer (Model No. 908-0015-0000) was used to determine the N_2_O concentration. We used the gas flux calculation formula from Song et al. (2003), and the specific calculation formula is as follows:

$$F = {\rm{\rho }} \cdot {V \over A} \cdot {{{P_S}} \over {{P_0}}} \cdot {{{T_0}} \over T} \cdot {{d{C_t}} \over {{d_t}}}$$

Where *F* is the gas flux (μg·m^−2^·h^−1^ or mg·m^−2^·h^−1^); ρ is the gas density at STP (standard temperature and pressure: CO_2_, CH_4_ and N_2_O: 1.977, 0.717 and 1.978 kg·m^−3^, respectively); *V* is the chamber volume (m^3^); *A* is the base area of the static chamber (m^2^); *P*_S_ is the atmospheric pressure (kPa) at the sampling sites; *P*_0_ is the atmospheric pressure at STP (101.325 kPa); *T*_0_ is the temperature at STP (273.15 K); *T* is the temperature inside the chamber (K), and *dC_t_*/*dt* is the rate of change in gas concentration over time.

The cumulative amount of CH_4_, CO_2_ and N_2_O emissions were sequentially determined from the emissions recorded from each set of two subsequent days. The effect of GHG emissions from herbicide use is expressed in terms of global warming potential (GWP or CO_2_ equivalent). The GWP of CH_4_ and N_2_O was founded to be 25 times and 298 times that of CO_2_, respectively, on the 100-year horizon. (Chen et al., 2011*; Liu et al., 2017*).

The aboveground biomass (g·m^−2^) of alfalfa and weeds was sampled in 2017 and 2018 using three randomly placed quadrats (1 m × 1 m) along a diagonal transect in each plot on day 0, day 2, day 6 and day 15 following the application of herbicides.

Three soil samples were taken from each plot at the time of gas collection using a soil drill with a diameter of 50 mm to depths of 0–10 cm. Fresh samples were used to determine the percentage of soil moisture (SM, %) (Song, 1993).

### Statistical analyses

Statistical analyses were conducted using SPSS 20.0 (SPSS, Inc., Armonk, NY, USA) with significance levels set at 0.05. Differences in the soil moisture, soil temperature, CH_4_, CO_2_ and N_2_O fluxes caused by the herbicides, days and application years were examined by ANOVA. The differences in the cumulative CH_4,_ CO_2_ and N_2_O emissions between the treatments and years were examined using Duncan’s new multiple range test and an independent-sample *t*-test, respectively. The analysis of interactions between the soil moisture, soil temperature, aboveground biomass of alfalfa and weeds and cumulative GHG emissions were examined using general linear models.

A structural equation model (SEM) was developed using IBM SPSS Amos version 17 (Amos Development Company, Greene, Maine, USA; SPSS Inc., Chicago, IL, USA), which was used to quantify the relationship between the treatments and GHG emissions. We considered all the endogenous variables, including treatments (QE, BT and CK), soil moisture, soil temperature and the aboveground biomass of alfalfa and weeds on the GHG fluxes (CO_2_, CH_4_ and N_2_O). The chi-square test was used to evaluate the fit of the model indicated by 0 ≤ χ^2^/df ≤ 2 and 0.05 < *P* ≤ 1.

## Results

### Soil moisture and soil temperature

**Figure 1:**
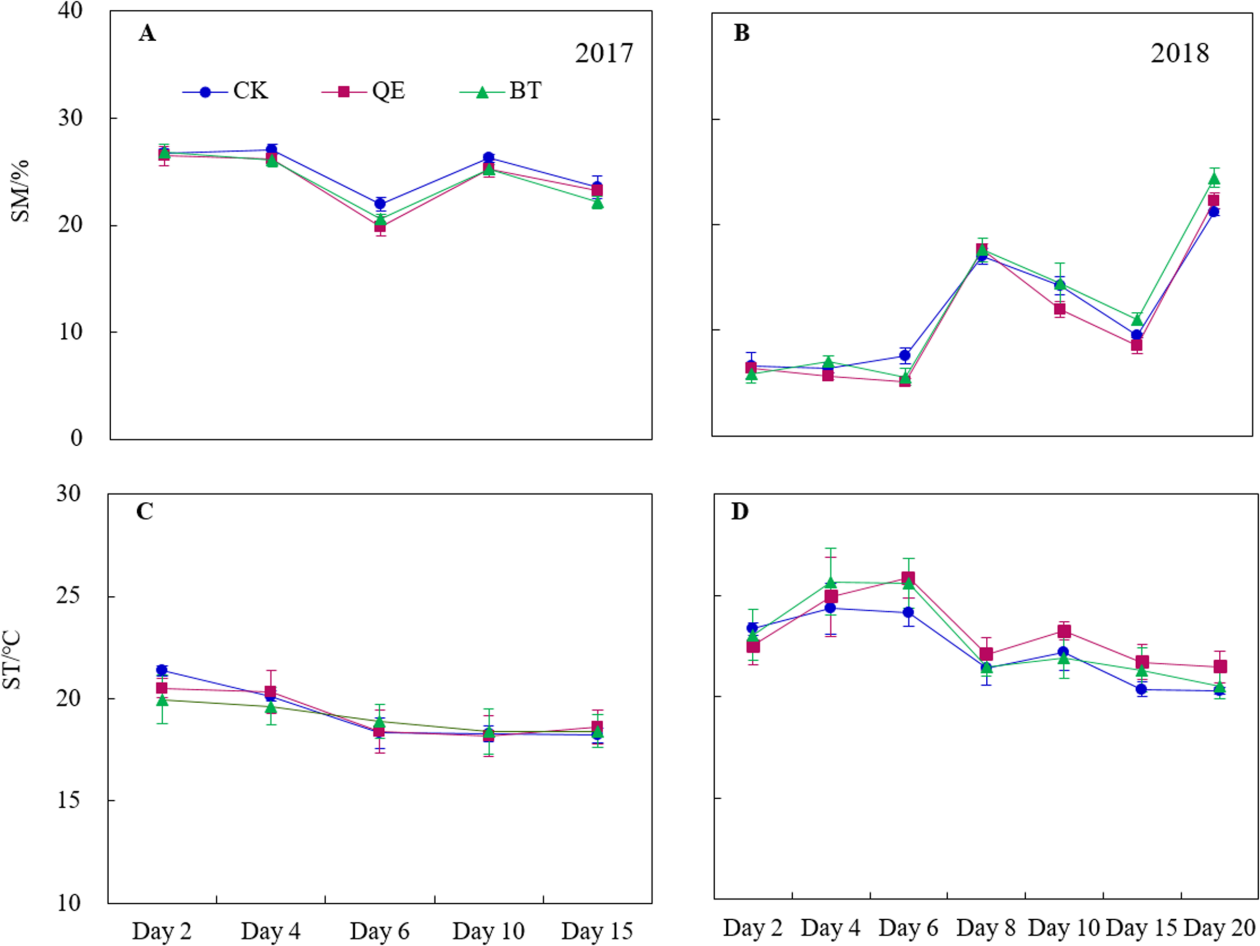
Daily dynamics of the soil moisture (SM) (A and B) and soil temperature (ST) (C and D) throughout the field trials in the two years (2017 and 2018). For the SM and ST, points with no mark are not significantly different between treatment in the same sampling time (*P* > 0.05).

The soil moisture content varied greatly throughout the field trials (Figs. 1A and 3B), however, no significant difference in soil moisture was observed between the treatments (*P* > 0.05) (Fig. 1B). Soil moisture increased on the day 10 in 2017 after a rainfall of 9.9 mm. High-intensity precipitation (28.4 mm on day 7 and 28 mm on day 19) in 2018 also resulted in an increase in moisture on days 9 and 20 of the herbicide applications when compared to days 6 and 15. There was no significant difference in soil temperature between the treatments (*P* > 0.05) (Figs. 1C and 1D).

There was a significant effect of the year, treatment, sampling time, the interaction between year and sampling time, the interaction between treatment and sampling time and interaction between year, treatment and sampling time for soil moisture (*P* < 0.05) (Table 1). There was a significant effect of the year, sampling time and interaction between year and sampling time for soil temperature (*P* < 0.05) (Table 1).

**Table 1:** The effects of interaction on the soil moisture and soil temperature.

Variable	Soil moisture		Soil temperature	
	*F*	*P*	*F*	*P*
*Y*	2,651.76	<0.001	117.82	<0.001
*T*	9.88	<0.001	1.01	0.37
*S*	181.75	<0.001	6.14	<0.001
*Y* × *T*	0.04	0.96	1.48	0.23
*Y* × *S*	64.69	<0.001	2.78	<0.05
*T* × *S*	3.05	<0.01	0.60	0.83
*Y* × *T* × *S*	3.70	<0.001	0.63	0.75

**Note:**

*Y*, year; *T*, treatment; *S*, sampling time; *Y* × *T*, interaction between year and treatment; *Y* × *S*, interaction between year and sampling time; *T* × *S*, interaction between year and sampling time; *Y* × *T* × *S*, interaction between year, treatment and sampling time.

### Aboveground biomass of alfalfa and weeds

**Figure 2:**
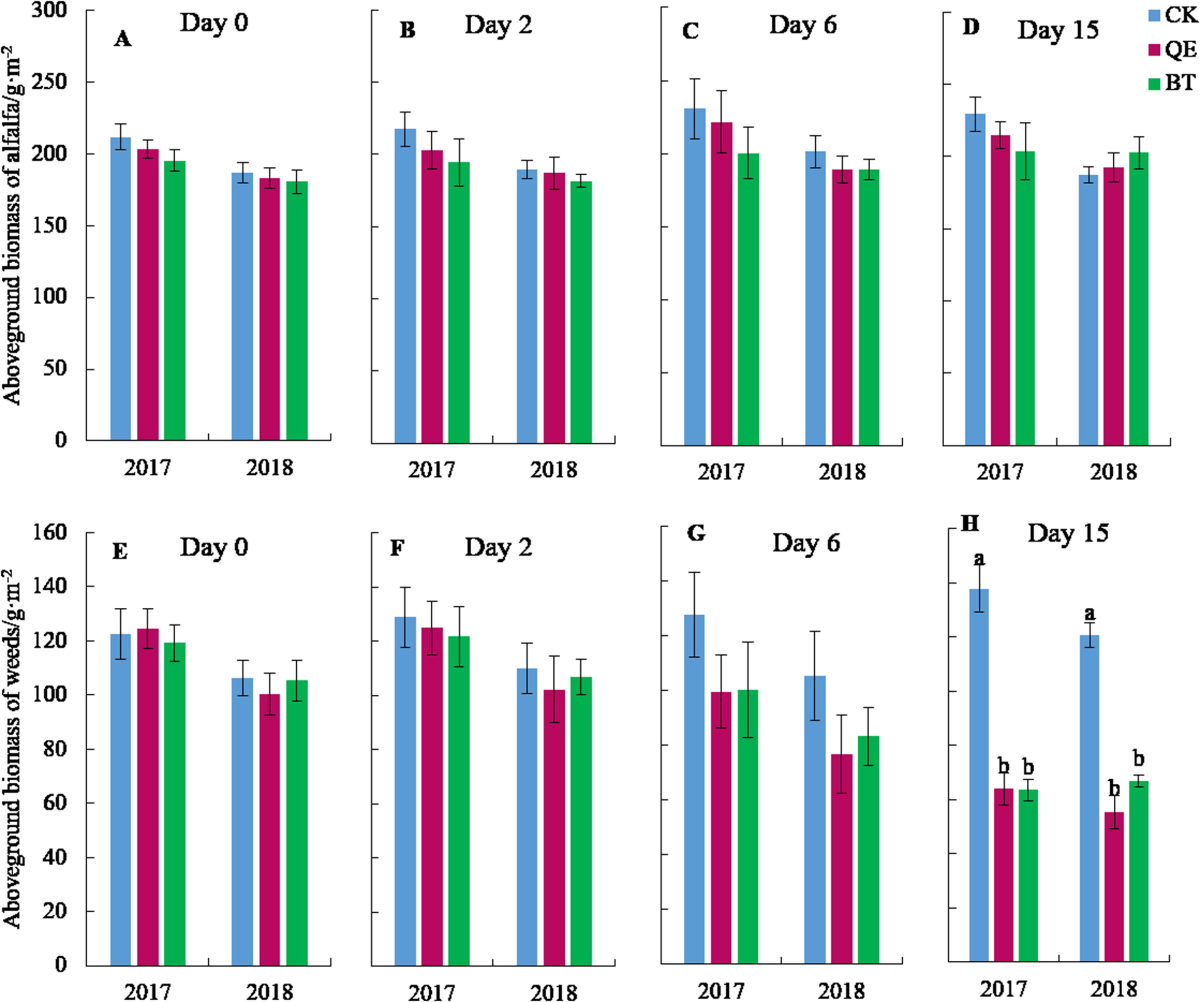
Daily dynamics of aboveground biomass of alfalfa (A–D) and weeds (E–H) at the sampling time after herbicide application in the two years (2017 and 2018). For the aboveground biomass of alfalfa (A–D), columns with no letters are not significantly different (*P* > 0.05). For the aboveground biomass of weeds (E–H), columns with no letters are not significantly different (*P* > 0.05), and with different lowercase letters are significantly different (*P* < 0.05).

There was no significant effect on aboveground alfalfa biomass following the application of herbicides (*P* > 0.05) (Figs. 2A–2D). The biomass of aboveground weeds was significantly reduced after the application of herbicides (*P* < 0.05) (Fig. 2H). There was no significant difference in the aboveground biomass of weeds between the CK and both herbicides on day 0, day 2 and day 6 following herbicide application (Figs. 2E–2G). The aboveground weed biomass decreased compared to the CK by 28.23% (*P* = 0.148) and 37.61% (*P* = 0.098) following the QE treatment on day 6 in 2017 and 2018, respectively. The aboveground weed biomass decreased compared to the CK by 27.44% (*P* = 0.186) and 26.64% (*P* = 0.074) following BT treatment on day 6 in 2017 and 2018, respectively. However, on day 15, after the application of QE, the aboveground weed biomass was significantly lower than that of the CK by 115.82% (*P* = 0.002) in 2017 and by 117.72% (*P* = 0.001) in 2018, whereas after the BT treatment, the aboveground weed biomass decreased by 117.0% (*P* = 0.002) in 2017 and 80.77% (*P* = 0.000) in 2018.

The year had a significant effect on the aboveground biomass of alfalfa. The year, treatment, sampling time, interaction between treatment and sampling time had significant effects on the aboveground biomass of weeds (Table 2).

**Table 2:** The effects of interaction on the aboveground biomass of alfalfa and weeds.

Variable	Alfalfa		Weeds	
	*F*	*P*	*F*	*P*
*Y*	4.750	<0.05	14.929	<0.001
*T*	0.861	0.431	29.695	<0.001
*S*	0.562	0.575	19.562	<0.001
*Y* × *T*	0.419	0.661	0.586	0.562
*Y* × *S*	0.221	0.803	1.045	0.362
*T* × *S*	0.204	0.934	8.807	<0.001
*Y* × *T* × *S*	0.058	0.994	0.150	0.962

**Note: **

*Y*, year; *T*, treatment; *S*, sampling time; *Y* × *T*, interaction between year and treatment; *Y* × *S*, interaction between year and sampling time; *T* × *S*, interaction between year and sampling time; *Y* × *T* × *S*, interaction between year, treatment and sampling time.

### GHG fluxes

**Figure 3:**
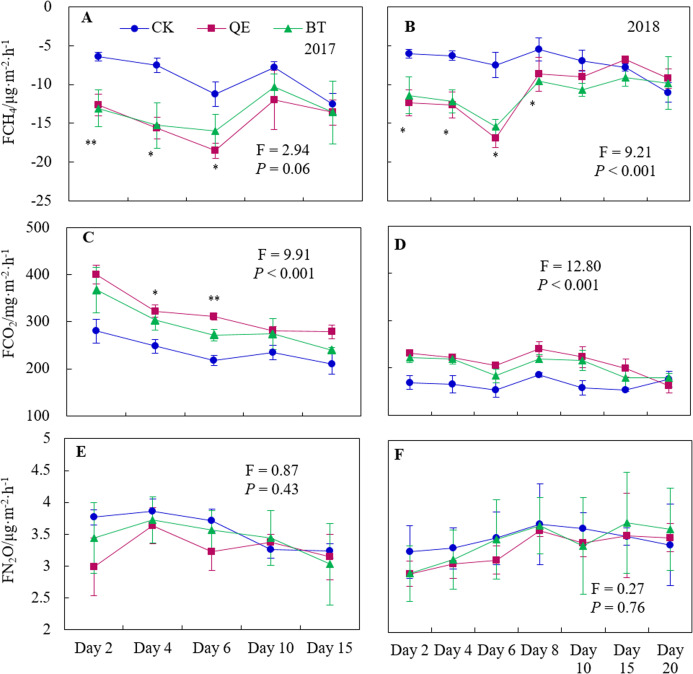
Daily dynamics of the CH_4_ fluxes (A and B), CO_2_ fluxes (C and D) and N_2_O fluxes (E and F) throughout the field trials in the two years (2017 and 2018) from soil planted with alfalfa. *Significant at *P* < 0.05; **Significant at *P* < 0.01.

The application of herbicides on alfalfa did not change the dynamics of CH_4_ uptake in the soil (Figs. 3A and 3B), but it significantly increased the capacity of soil to absorb CH_4_ (*P* < 0.05). CH_4_ fluxes all decreased significantly within 6 days and 8 days following the QE and BT treatments in 2017 and 2018, respectively (*P* < 0.05), compared with the CK. There was no significant difference shown between the other sampling times. Peak CH_4_ uptake occurred on day 6 in 2017 and 2018 after the herbicide applications and there was no significant difference in CH_4_ fluxes between the QE and BT treatments in both years (*P* > 0.05).

Significantly more CO_2_ emissions were recorded following the application of QE and BT than in the CK (Figs. 3C and 3D). CO_2_ emissions were significantly higher on days 4 and 6 in 2017 after the herbicide application than in the CK (*P* < 0.05). In 2018, the CO_2_ fluxes were significantly higher than those of the CK within 8 days after the herbicide applications (*P* < 0.05). The application of QE and BT had no significant influence on CO_2_ emissions relative to the CK in the subsequent samples.

Herbicide applications did not change the dynamics of N_2_O emissions from the soil beneath the alfalfa (Figs. 3E and 3F). N_2_O fluxes were lower than those of the CK in the first 6 days after herbicide application in 2017 but the difference was not significant (*P* > 0.05). The N_2_O emissions from 2018 were lower for the first 4 days after the herbicide treatments than those in the CK, but this difference was not significant. There were no significant differences in N_2_O fluxes between treatments (*P* > 0.05) for the remaining sampling time.

### GHG cumulative emissions during the sampling times and the integrated evaluation of GHG emissions

There was no significant difference in the cumulative uptake of CH_4_ between years in all three treatments (*P* > 0.05) (Table 3). In 2017, the cumulative CH_4_ uptake following the application of QE and BT increased by 59.63% (*P* = 0.002) and 50.46% (*P* = 0.003), respectively, compared with those in the CK and were ordered as: QE > BT > CK. In 2018, the cumulative CH_4_ uptake increased by 47.97% (*P* = 0.002) and 52.85% (*P* = 0.007) following the application of QE and BT, respectively, compared to the CK, and were ordered as: CK > QE > BT.

**Table 3:** Effects of herbicide applications on the cumulative GHG emissions during the sampling times, the GWP of soil planted with alfalfa, and the effect of interaction on cumulative GHG emissions and GWP.

Year	Treatments	CH_4_ (g·ha^−1^)		CO_2_ (kg·ha^−1^)		N_2_O (g·ha^-1^)		Total GWP	
2017	CK	−10.94 ± 0.52Aa		285.71 ±11.96Ac		4.28 ± 0.04Ba		286.71 ± 11.96Ac	
	QE	−17.36 ± 0.76Ab		382.25 ± 4.29Aa		3.93 ± 0.16Ba		382.99 ± 4.31Aa	
	BT	−16.37 ± 0.92Ab		349.38 ± 4.10Ab		4.13 ± 0.13Ba		350.20 ± 4.08Ab	
	F	21.165		40.521		2.100		40.312	
	*P*	<0.01		<0.001		0.204		<0.001	
2018	CK	−12.27 ± 0.57Aa		285.55 ± 5.81Ab		5.81 ± 0.15Aa		286.97 ± 5.87Ab	
	QE	−18.17 ± 0.59Ab		355.77 ± 2.40Ba		5.54 ± 0.06Aa		356.97 ± 2.41Ba	
	BT	−18.79 ± 1.13Ab		340.57 ± 5.83Aa		5.73 ± 0.31Aa		341.81 ± 5.88Aa	
	F	20.058		55.705		0.449		54.420	
	*P*	<0.01		<0.001		0.658		<0.001	
**Variable**		**CH_4_**		**CO_2_**		**N_2_O**		**GWP**	
		***F***	***P***	***F***	***P***	***F***	***P***	***F***	***P***
*Y*		13.099	<0.01	160.459	<0.001	0.368	0.546	4.608	0.053
*T*		18.303	<0.001	33.574	<0.001	0.649	0.526	86.830	<0.001
*S*		5.562	<0.001	8.363	<0.001	0.465	0.832	–	–
*Y* × *T*		0.187	0.830	1.649	0.199	0.022	0.978	2.126	0.162
*Y* × *S*		0.800	0.529	8.782	<0.001	1.224	0.308	–	–
*T* × *S*		1.938	<0.05	0.986	0.471	0.169	0.999	–	–
*Y* × *T* × *S*		0.272	0.973	0.332	0.951	0.055	1.000	–	–

**Note: **

*Y*, year; *T*, treatment; *S*, sampling time; *Y* × *T*, interaction between year and treatment; *Y* × *S*, interaction between year and sampling time; *T* × *S*, interaction between year and sampling time; *Y* × *T* × *S*, interaction between year, treatment and sampling time. Different lowercase letters represent significant differences between the treatments at *P* < 0.05 in the same year, and different capital letters represent significant differences between the years at *P* < 0.05 in the same treatment. GWP, global warming potential.

Aggregate CO_2_ emissions from both years were: QE > BT > CK (Table 3). The cumulative CO_2_ emissions following treatment with QE in 2017 were significantly higher than those in 2018 (*P* = 0.006). In 2017, the cumulative CO_2_ emissions following the application of QE and BT were significantly higher than those of the CK (34.15% (*P* = 0.002) and 22.27% (*P* = 0.007), respectively). In 2018, cumulative CO_2_ emissions in the plots treated with QE and BT were 24.59% (*P* = 0.000) and 19.27% (*P* = 0.003) above those of the CK, respectively.

The cumulative N_2_O fluxes were: CK > BT > QE in 2017 and 2018 (Table 3). In 2017, the cumulative N_2_O emissions following the application of QE and BT were below those of the CK (8.73% (*P* = 0.106) and 3.68% (*P* = 0.313), respectively). In 2018, the cumulative N_2_O emissions following the application of QE and BT were below those of the CK (4. 63% (*P* = 0.169) and 1.50% (*P* = 0.818), respectively).

The global warming potential (GWP), expressed as the 100-year total CO_2_ equivalent, was used to comprehensively evaluate the GHG emissions from different herbicide treatments. The application of QE and BT significantly increased GWP by 25.14% (*P* = 0.002) and 22.15% (*P* = 0.007) compared with the CK in 2017, respectively. In 2018, the application of QE and BT resulted in a significant increase of 24.39% (*P* = 0.000) and 19.11% (*P* = 0.003), respectively, relative to the CK. A significant difference in the GWP was recorded following the application of QE in 2017 and 2018 (Table 3).

The year, treatment and sampling time had significant effects on CH_4_ and CO_2_ fluxes (Table 3). The interaction between year and treatment had a significant effect on the CO_2_ fluxes, as did the interaction between treatment and sampling time.

### Structural equation model

**Figure 4:**
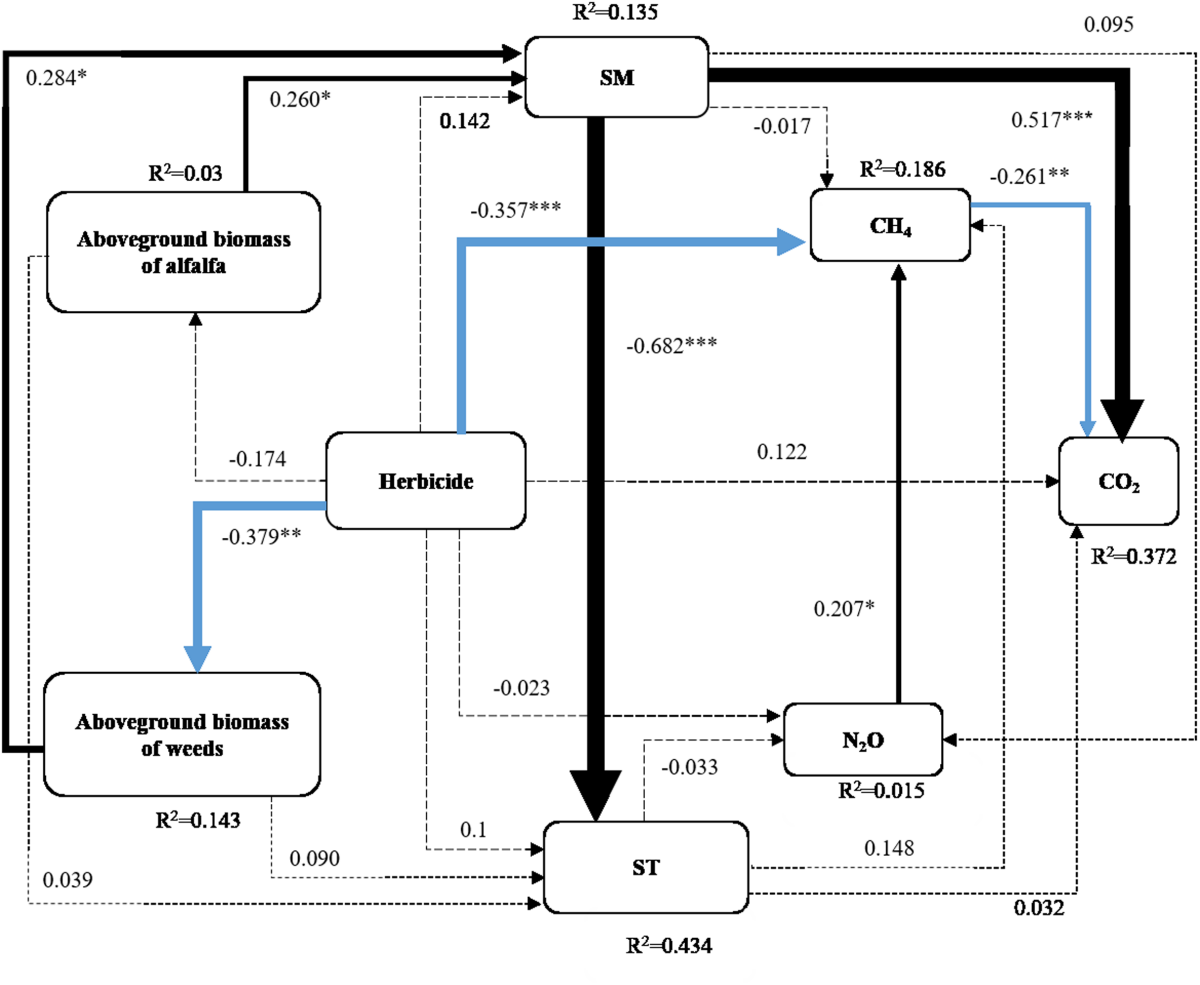
Structural equation model based on the data in this study. Structural equation modeling shows the effects of herbicide treatments on soil moisture (SM), soil temperature (ST), CH_4_ fluxes, CO_2_ fluxes and N_2_O fluxes. Solid and dotted lines represent the direct and indirect effects, respectively. The arrows reflect the causal relationships, and the thickness of the black (positive) and blue (negative) paths is proportional to the range-standardized path coefficients with significance levels indicated as ***P < 0.001; **P < 0.01, and *P < 0.05. Insignificant paths are indicated by dashed arrows (*P* > 0.05). The model shows a fitted result (χ^2^ = 4.959, df = 8,*P* = 0.762).

A SEM was used to analyze direct and indirect relationships among multiple variables. The direct effects of herbicide applications on the aboveground biomass of weeds and CH_4_ fluxes were highly significant (Fig. 4) (*P* < 0.01). The aboveground biomass of the alfalfa and weeds had direct positive effects on the soil moisture (*P* < 0.05). The soil moisture had an extremely significant and direct effect on the soil temperature and CO_2_ fluxes (*P* < 0.001). There was an extremely significant and direct positive effect on the CH_4_ fluxes through N_2_O fluxes (*P* < 0.05), and CH_4_ fluxes had a significant and direct negative effect on CO_2_ fluxes. The SEM results show that herbicides may affect CH_4_ uptake from alfalfa pasture and affect the greenhouse soil gas emissions by indirectly affecting the aboveground biomass of vegetation and environmental factors, such as soil moisture and soil temperature.

## Discussion

### Effect of herbicides on methane fluxes in the soil of alfalfa pastures

Our field study demonstrated that CH_4_ uptake was enhanced following the application of QE and BT (Figs. 3A and 3B), which is similar to the increase in CH_4_ uptake as a result of the application of Prosulfuron observed by Kinney et al. (2004b). Soil treated by herbicides maintained a relatively higher oxidation-reduction potential than those of the CK, which resulted in smaller CH_4_ fluxes following the application of QE and BT and increased CH_4_ uptake by pasture soil. Mohanty et al. (2001) demonstrated that oxidation–reduction potential is negatively correlated with the CH_4_ fluxes. Therefore, increased CH_4_ uptake after the application of herbicides is due to the advance of the soil oxidation–reduction process (Das, Ghosh & Adhya, 2011*)*.

Soil temperature affects CH_4_ emissions primarily by affecting the soil microbial activity and gas diffusion rates (Bates et al., 2009). CH_4_ fluxes were indirectly affected by the application of herbicides (Fig. 4). CH_4_ uptake may increase with higher soil temperatures in arid areas due to the limited ability of soil microorganisms to oxidize CH_4_ (Fang et al., 2009). However, when one or more environmental factors limit the formation of CH_4_ this effect is not manifested (Chen et al., 2011). Our research found that soil moisture had significant and direct effects on CH_4_ fluxes and soil moisture was significantly and directly affected by the aboveground biomass of alfalfa and weeds (Fig. 4). The application of herbicides reduced the aboveground biomass of weeds (Fig. 2H), leading to more exposed surface area, an increase in soil moisture evapotranspiration, and a decrease in soil moisture (Jia et al., 2007). The activity of CH_4_ oxidizing bacteria was inhibited and the uptake of CH_4_ was increased (Jiao et al., 2014). CH_4_ fluxes were significantly and directly affected by N_2_O fluxes (Fig. 4). N_2_O is generated by denitrification and nitrification and the anaerobic conditions of denitrification aid the activity of CH_4_ oxidizing bacteria, thereby resulting in the increases of the CH_4_ fluxes (Dang et al., 2015).

### Effect of herbicides on carbon dioxide fluxes in the soil of alfalfa pastures

The application of QE and BT significantly increased CO_2_ emissions (Figs. 3C and 3D). This effect may be related to the selective herbicide use in pastures that causes the partial necrosis of pasture weeds, transforming them into litter (Fig. 2H). The carbon input from the pasture litter increased following herbicide treatments compared with that of the CK, which provided a carbon source for soil microbial activities, increasing in CO_2_ emissions (Figs. 3C and 3D) (Zheng et al., 2018). Alternatively, weed growth was inhibited by the use of herbicides (Fig. 2H) and the interspecific competition between the weeds and alfalfa was reduced, allowing water into the soil to be absorbed and utilized by the alfalfa, resulting in increased soil moisture following herbicide treatments compared with the CK (Figs. 1A and 1B), and promoting CO_2_ emissions from the soil (Fig. 4). These findings are consistent with those of Wang et al. (2013). The application of QE and BT can also activate urease activity, leading to the production of CO_2_ by catalyzing the decomposition of urea in the soil (Sannino & Gianfreda, 2001).

CO_2_ emissions from grassland ecosystems originated primarily from soil respiration, which includes respiration by plant roots and microorganisms and the CO_2_ produced by the microbial decomposition of organic matter (Yevdokimov et al., 2010). Soil moisture had a significant and direct effect on CO_2_ emission (Fig. 4). Soil CO_2_ emissions increased with the increase in soil moisture within a certain range and were the highest when soil moisture was close to field capacity (Yu & Fang, 2010). Respiration stagnated at both the saturation and permanent wilting points (Vargas et al., 2014). CO_2_ fluxes were significantly and directly affected by CH_4_ (Fig. 4). A previous study indicated that CH_4_ absorbed by the soil can provide a carbon source for CO_2_ emissions (Peng et al., 2015).

### Effect of herbicides on global warming potential in the soil of alfalfa pastures

We demonstrated the potential importance of herbicide applications on the modulation of GWP relative to soils, climate, and other agricultural practices (Kinney et al., 2004a). The application of two herbicides significantly affected the fluxes of CH_4_ and CO_2_. However, the most important increase in total GWP was caused by an increase in the flux of CO_2_, which accounted for the majority of increased GWP (Table 3). Herbicide use could be responsible for some of the variation in soil gas fluxes (Kinney et al., 2004b). Temperature, precipitation and other agricultural practices over time may lead to inter-annual variations in GWP following QE (Shang et al., 2016).

The global use of agrochemicals was expected to rise 2.7 times over the next 50 years, owing to their irrefutable economic benefits in agricultural practices (Jiang et al., 2015). Different herbicides vary in their effects on greenhouse gas emissions due to differences in their mechanism of action and molecular structures. The actual effects of the combined application of herbicides on GHG fluxes of alfalfa pastures may differ from the results of this study. Therefore, the effects of widely used herbicides on GHG fluxes must be studied to determine the best combination of herbicides to ensure crop yields and reduce the greenhouse effect. Additional studies should evaluate the risks to soil environmental associated with herbicide contamination.

## Conclusions

This field study demonstrates that the application of herbicides on alfalfa increases soil GHG emissions. The application of Quizalofop-p-ethyl and BT had significant effects on increasing soil CH_4_ uptake and soil CO_2_ emissions, but it did not affect N_2_O emissions. Our results showed that the effect of Quizalofop-p-ethyl was greater than that of BT on GHG emissions. Additional studies should focus on separate applications of the herbicides Quizalofop-p-ethyl and BT to determine the mechanisms that govern the observed increases in the CH_4_ uptake and CO_2_ emissions. Furthermore, our study suggests that herbicide use is vital when considering the effect of agricultural practices on GHG fluxes.

## Supplemental Information

10.7717/peerj.9231/supp-1 Supplemental Information 1 Changes of aboveground biomass, soil moisture and temperature and greenhouse gas.

## Additional Information and Declarations

### Data Availability

The following information was supplied regarding data availability: The raw measurements are available in the Supplemental File.
